# Hypomagnesemia Caused by Chronic Use of Over-the-Counter Proton Pump Inhibitor as a Possible Cause of Supraventricular Tachycardia

**DOI:** 10.7759/cureus.42606

**Published:** 2023-07-28

**Authors:** Francisco J Ortega, Frederick N Campos, Branidia Mercedes

**Affiliations:** 1 Internal Medicine, Wyckoff Heights Medical Center, Brooklyn, USA

**Keywords:** electrolytes and arrhythmias, cardiac arrhythmia, proton pump inhibitor, atrial tachycardia, hypomagnesemia, supraventricular tachycardia (svt)

## Abstract

Magnesium is an important co-factor that helps regulates the movement of ions through voltage-mediated channels within myocardial tissues by the membrane sodium-potassium pump, and its deficiency can reduce the pump’s activity, leading to partial depolarization and changes in the activity of many potential-dependent membrane channels leading to arrhythmias. In this case report, we are looking to establish the direct relationship between hypomagnesemia caused by proton pump inhibitors (PPIs), which could lead to cardiac arrhythmias. Here, we present a 45-year-old Hispanic female, with a known past medical history of supraventricular tachycardia (SVT), hiatal hernia on proton pump inhibitor (PPI), and chronic smoking, who presented to the emergency department complaining of dizziness and palpitations that started two hours prior arrival to the hospital. At triage, the patient was found to have a heart rate of 190 beats per minute (bpm), and an electrocardiogram (EKG) revealed supraventricular tachycardia with a heart rate of 185 bpm. During the review of this case, no other confounding factors besides hypomagnesemia were noted, leaving this one to be the most likely cause of the arrhythmia. Patients on long-term PPI therapy are at higher risk of developing hypomagnesemia, which could lead to cardiac arrhythmia.

## Introduction

Magnesium is an important co-factor that helps regulates the movement of ions through voltage-mediated channels within myocardial tissues by the membrane sodium-potassium pump, and its deficiency can reduce the pump’s activity, leading to partial depolarization and changes in the activity of many potential-dependent membrane channels leading to arrhythmias. Magnesium plays an essential role in many fundamental biological reactions, as it is involved in more than 300 metabolic reactions. A deficiency of magnesium may result in many disorders, including cardiac arrhythmias. Moreover, magnesium ions play a crucial role in the functioning of many ion channels, including cardiac Mg2+ sensitive K+ channels, which normally allow K+ to pass more readily inward than outward. As magnesium regulates the outward K+ movement, potassium is transported equally well in both directions when Mg2+ is absent. Therefore, magnesium deficiency may lead to a reduced amount of intracellular K+, which disturbs the resting membrane potential of the heart muscle cells and results in cardiac arrhythmias, such as supraventricular tachycardia (SVT).

Supraventricular tachycardia is a term used to describe tachycardias (atrial and/or ventricular rates more than 100 bpm at rest), the mechanism of which involves tissue from the His bundle or above. SVT terms include inappropriate sinus tachycardia, atrial tachycardia (AT) (including focal and multifocal AT), macroreentrant AT (including typical atrial flutter), junctional tachycardia, atrioventricular nodal reentrant tachycardia (AVNRT), and various forms of accessory pathway-mediated reentrant tachycardia [1]. SVTs are caused by an anomaly in the conduction electrical system of the heart. SVT manifestations are random and paroxysmal but can be triggered by events, substances (alcohol, coffee, and tea), underlying conditions (chronic obstructive pulmonary disease, hyperthyroidism, and pregnancy), recreational drugs (amphetamines), smoking, congenital heart disease (Wolff-Parkinson-White syndrome), exertion, electrolyte imbalance, and more.

Herein, in this publication, we are looking to highlight the risk of developing cardiac arrhythmias, such as SVT, caused by hypomagnesemia related to prolonged proton pump inhibitor (PPI) intake and the necessity of monitoring magnesium levels in this population.

## Case presentation

A 45-year-old Hispanic female, with a known past medical history of SVT, hiatal hernia, and chronic smoking, presented to the emergency department complaining of palpitations, dizziness, and shortness of breath that started approximately two hours prior to arrival to the hospital while walking in the train station. The patient also reported light-headedness and severe palpitation, associated with shortness of breath. The symptoms lasted approximately 30 minutes, which prompted her to come to the hospital. The patient reported being diagnosed with some type of cardiac arrhythmia in the past for which she was evaluated by a cardiologist in Florida two years ago. She stated that she had intermittent episodes of palpitations in the past. She reported exertional dyspnea after walking 1.5 blocks or going uphill. The patient denied recent alcohol use, recreational drugs, and coffee intake but reported daily tea intake, every 4-6 hours, 10-15 tea bags per drink. The patient denied any known drug allergies, smokes 1-2 cigarettes per day, and drinks around four drinks of vodka on “some” Saturdays. The patient only reported omeprazole as her medication for her heartburn symptoms at home.

**Figure 1:**
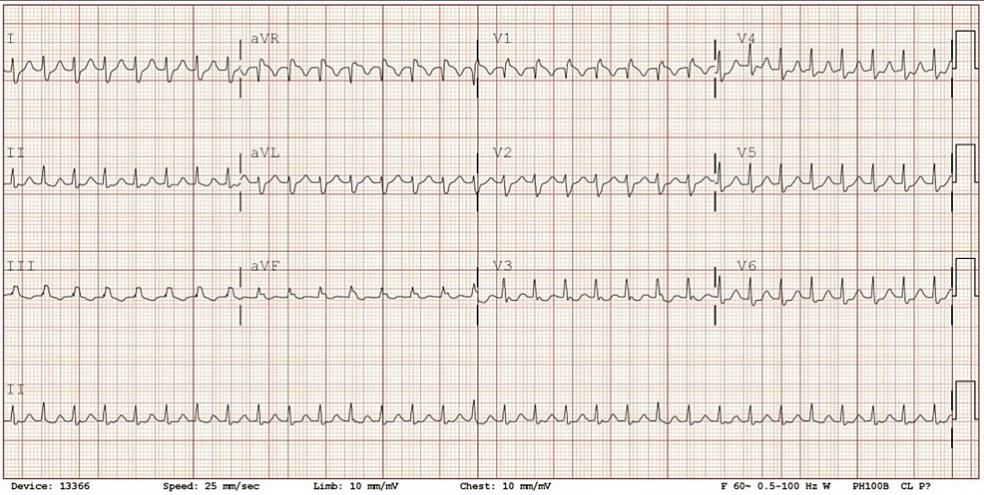
Electrocardiogram of the patient upon arrival at the emergency department.

At triage, her temperature was 36.7°C, blood pressure was 156/137 millimeters of mercury (mmHg), heart rate (HR) was 195 beats per minute (bpm), respiratory rate was 20 breaths per minute, and oxygen saturation was 99% on room air. During the emergency department evaluation, the electrocardiogram (EKG) showed an HR of 180 bpm (narrow complex tachycardia) (Figure 1). No ST segment changes are suggested for ischemia. A vagal maneuver was attempted but failed to break the arrhythmia, followed by the administration of Cardizem 20 milligrams (mg) intravenously pushed, which broke the SVT. The patient did not meet systemic inflammatory response syndrome (SIRS) criteria during the emergency department evaluation, and she was admitted to the telemetry unit for further monitoring.

While admitted to the telemetry unit, triggers for SVT episodes were investigated. The thyroid panel was normal. Urine toxicology and caffeine levels were negative. Complete blood count, creatinine phosphokinase, and complete metabolic panel were within normal limits. Chest X-ray showed small airway disease without lobar consolidation. The magnesium level was 0.9 mg/dL. Magnesium was supplemented with a total of 4 g IV. Initial troponin was negative (24.4 ng/L), trended up to 111 ng/L, and then trended down to 47.1 ng/L. On the second day of hospitalization, the patient continued to remain at sinus rhythm on the cardiac monitor, and no new events were recorded. The cardiology service evaluated the patient, recommended starting her on metoprolol tartrate 25 mg twice a day, and advised reducing caffeine intake to obtain an echocardiogram, which reported normal left ventricular cavity size, wall thickness, and systolic function. The patient had an ejection fraction of 55%-60%. The structure of the mitral, aortic, and tricuspid valves was normal. Mild regurgitation was reported on mitral and tricuspid valves. The patient was also evaluated by the electrophysiology service, who advised her to follow up as an outpatient for evaluation on possible cardiac ablation. Troponin elevation was attributed to demand ischemia on the setting of tachycardia. No signs of infections were noted during hospitalization. The patient was discharged on day 3 of hospitalization and was advised to follow up with the cardiology clinic.

## Discussion

As previously mentioned, supraventricular tachycardia is a group of arrhythmias originating from the atrioventricular node or above of it. Many external or internal factors can trigger supraventricular tachycardia, such as tobacco, electrolyte abnormalities, exertion, caffeine, stress, alcohol, and medications.

Magnesium is an important co-factor that helps regulates the movement of ions through voltage-mediated channels within myocardial tissues by the membrane sodium-potassium pump, and its deficiency can reduce the pump’s activity, leading to partial depolarization and changes in the activity of many potential-dependent membrane channels leading to arrhythmias.

The clinical case presented in this report portrays a middle-aged Hispanic female who was admitted for an episode of supraventricular tachycardia. The culprit for triggering her arrhythmia seems to be the hypomagnesemia of 0.9 mg/dL noted during the emergency department evaluation, which is probably related to the constant intake of over-the-counter proton pump inhibitors (PPIs) for her heartburn symptoms, caused by her hiatal hernia. PPI use has been demonstrated to cause magnesium (Mg2+) deficiency (hypomagnesemia). PPI-induced hypomagnesemia is associated with clinical complaints including fatigue, muscle cramps, and arrhythmias. In general, PPI-induced hypomagnesemia occurs during long-term PPI treatment (>1 year). Mg2+ absorption in the small intestine depends on passive paracellular diffusion. Consequently, two factors are essential to consider: Mg2+ availability and tight junction permeability. Both factors are potentially compromised by PPI treatment [2]. Magnesium ions play a crucial role in the functioning of many ion channels, including cardiac Mg2+ sensitive potassium (K+) channels, which normally allow K+ to pass more readily inward than outward. As magnesium regulates the outward K+ movement, potassium is transported equally well in both directions when Mg2+ is absent. Therefore, magnesium deficiency may lead to a reduced amount of intracellular K+, which disturbs the resting membrane potential of the heart muscle cells and results in cardiac arrhythmias [3].

Although the patient was exposed to other factors that could be presented as confounding bias, such as the fact that the patient has a known history of underlying arrhythmias, chronic smoker status, and daily use of tea in a significant amount, none of these elements were likely to be the determining trigger for this episode. According to the Food and Drug Administration (FDA), there is 30-50 mg of caffeine in each cup of green or black tea [4]. The patient’s level of caffeine upon arrival at the hospital was negative. The patient has also been a chronic light smoker for many years, and she did not report an increase in the number of cigarettes recently prior to arrival at the hospital. Other causes that could have triggered the SVT, such as recreational drugs, thyroid disease, and other electrolyte abnormalities, were ruled out. No signs of infection were noted either. The only identifiable and reasonable trigger factor was hypomagnesemia secondary to PPI.

During the hospitalization course, the patient was started on metoprolol tartrate 25 mg twice a day by the cardiology department. No further runs of SVTs were noted on the cardiac monitor. The patient was advised to follow up with the electrophysiology clinic to explore options for treatments on ablation.

According to the American Heart Association, recommendations for treatment options (including drug therapy, ablation, or observation) must be considered in the context of the frequency and duration of the SVT, along with clinical manifestations, such as symptoms or adverse consequences (e.g., development of cardiomyopathy) [5].

## Conclusions

Proton pump inhibitors are helpful for gastric reflux symptoms and are available over the counter. This provides easy access to the general population without the necessity of a physician. Hypomagnesemia induced by chronic use of PPI is a rare but serious side effect that could lead to fatal cardiac arrhythmia. Magnesium is an important co-factor that helps in regulating voltage-mediated channels within myocardial tissues by the membrane sodium-potassium pump, and its deficiency can reduce the pump’s activity, leading to partial depolarization and changes in the activity of many potential-dependent membrane channels leading to arrhythmias.

It is important to educate patients on the chronic use of medication such as PPI without constant monitoring, as well as having a high amount of suspicious as a physician for hypomagnesemia in patients with chronic use of PPI, implementing periodic monitoring of magnesium levels during routine follow-up or discontinuation of this medication when not required, in addition to advising patients on not taking them without proper medical supervision.
